# Field‐grown soybean transcriptome shows diurnal patterns in photosynthesis‐related processes

**DOI:** 10.1002/pld3.99

**Published:** 2018-12-04

**Authors:** Anna M. Locke, Rebecca A. Slattery, Donald R. Ort

**Affiliations:** ^1^ Soybean and Nitrogen Fixation Research Unit USDA‐ARS Raleigh North Carolina; ^2^ Department of Crop and Soil Sciences North Carolina State University Raleigh North Carolina; ^3^ Carl R. Woese Institute for Genomic Biology University of Illinois Urbana Illinois; ^4^ Global Change and Photosynthesis Research Unit USDA‐ARS Urbana Illinois; ^5^ Department of Plant Biology University of Illinois Urbana Illinois

**Keywords:** circadian rhythm, *Glycine max*, photosynthesis, RNA‐seq, soybean, transcriptome

## Abstract

Many plant physiological processes have diurnal patterns regulated by diurnal environmental changes and circadian rhythms, but the transcriptional underpinnings of many of these cycles have not been studied in major crop species under field conditions. Here, we monitored the transcriptome of field‐grown soybean (*Glycine max*) during daylight hours in the middle of the growing season with RNA‐seq. The analysis revealed 21% of soybean genes were differentially expressed over the course of the day. Expression of some circadian‐related genes in field‐grown soybean differed from previously reported expression patterns measured in controlled environments. Many genes in functional groups contributing to and/or depending on photosynthesis showed differential expression, with patterns particularly evident in the chlorophyll synthesis pathway. Gene regulatory network inference also revealed seven diurnally sensitive gene nodes involved with circadian rhythm, transcription regulation, cellular processes, and water transport. This study provides a diurnal overview of the transcriptome for an economically important field‐grown crop and a basis for identifying pathways that could eventually be tailored to optimize diurnal regulation of carbon gain.

## 1 INTRODUCTION

Diurnal changes in plant physiological responses and gene expression are governed by diurnally changing environmental conditions and endogenous circadian rhythms. Coordinating physiological functions with the 24‐h clock allows plants to balance energy needs and resources (Dodd et al., 2005) and creates temporal compartments to prevent futile biochemical cycles. In C_3_ plants, ATP and NADPH are produced through the light reactions to fuel carbon assimilation in the C_3_ cycle only during daylight. Stomatal opening is stimulated by light to allow CO_2_ to enter the leaf (Kinoshita et al., 2001), which means transpiration occurs almost entirely in the light, driving water flow through the plant. Water flow into the roots in turn drives nutrient uptake from the soil. During the daytime, extra carbon must be assimilated to fuel respiration for cellular processes in the dark. This extra carbon is typically stored as starch in the chloroplast, resulting in diurnal cycles of starch accumulation and depletion (Stitt & Zeeman, 2012; Zeeman, Smith, & Smith, 2007).

Environmental conditions change greatly even within the light period, as plants in the field experience fluctuations in light quality, light intensity, temperature, wind, and vapor pressure deficit on a daily basis. Photosynthetic processes continually acclimate throughout the day to optimize light use efficiency and to maintain a balance between energy from the light reactions and substrates for the carbon reactions (Geiger & Servaites, 1994; Reinbothe & Reinbothe, 1996). Because light and temperature tend to peak in the middle of the day, it is likely that expression of photosynthesis‐related genes follows characteristic patterns over the course of the light period. Some photosynthesis‐related processes have been linked to expression of circadian rhythm‐related genes. For example, altered expression of circadian‐related genes in Arabidopsis (*Arabidopsis thaliana*) and maize (*Zea mays*) has been linked to increased rates of photosynthesis in hybrids (Bendix, Marshall, & Harmon, 2015; Ko et al., 2016).

Soybean (*Glycine max*) typically shows strong diurnal variations in photosynthesis, carbohydrate metabolism, and nitrogen assimilation (Bernacchi et al., 2006; Delhon, Gojon, Tillard, & Passama, 1995; Rogers et al., 2004; Upmeyer & Koller, 1973). Because carbon accumulation depends not just on peak midday photosynthesis, but on the daily integral of photosynthesis, the regulation of diurnal metabolic fluctuations impacts season‐long yield. Understanding the transcriptional basis of diurnal photosynthetic cycles could provide insight into how carbon assimilation is, or could be, maximized over the entire day, rather than just at peak photosynthesis. Based on known fluctuations in photosynthesis, sugar metabolism, and environmental conditions, we hypothesized that genes related to photosynthetic function and sugar metabolism would likely show diurnal changes in transcription.

Using RNA‐seq, this study examined diurnal changes in the leaf transcriptome of field‐grown soybean. We first compared circadian‐related gene expression in the field setting to previously measured expression patterns in controlled environments. We then identified diurnally regulated genes involved in various aspects of photosynthesis. Finally, we identified soybean genes integral to diurnal function in soybean.

## 2 MATERIALS AND METHODS

### 2.1 Plant material and growth conditions

For RNA‐seq analyses, soybean (*Glycine max*) cv. 93B15 (Pioneer Hi‐Bred, Johnston, IA) was planted on 27 May 2010 at the SoyFACE research farm near Champaign, Illinois (40.042° N, 88.237° W). Four plots in the field were the four biological replicates. Each replicate was sampled at four time points on the same day, and each replicate was sampled on a different day: 14, 16, 18, or 24 August 2010. All plants were in early to mid‐seed fill (R5‐R6) during this period. Day length during this time period ranged from 13:23 to 13:47 h. This experimental design accounted for environmental variation among sampling days with the replicate in the statistical model, distributed equally across time points. All three leaflets from an uppermost, fully expanded leaf were detached and immediately flash‐frozen in liquid nitrogen at 8:00 (zeitgeber time [ZT] 2:20), 11:00 (ZT5:20), 14:00 (ZT8:20), and 17:00 (ZT11:20), and subsequently stored at −80°C. The three leaflets were combined to create one sample per plot.

For diurnal measurements of chlorophyll content, leaf disks from 10 randomly selected plants were harvested at 8:00/ZT2:20, 11:00/ZT5:20, 14:00/ZT8:20, and 17:00/ZT11:20. These samples were harvested at SoyFACE on 16 August 2013 from cv. 93Y40 (Pioneer Hi‐Bred, Johnston, IA). Samples were cut from the uppermost, fully expanded leaf and immediately flash‐frozen in liquid nitrogen.

Climate data (temperature, relative humidity [RH], and photosynthetic photon flux density [PPFD]) for 30 min intervals throughout each sampling day were obtained from the University of Illinois Energy Farm weather station located 3.7 km northeast of the SoyFACE site.

### 2.2 RNA extraction and sequencing

Total RNA was isolated from whole leaflets with a method developed specifically for field‐grown soybean (Bilgin, DeLucia, & Clough, 2009), and RNA was treated with the DNA‐free kit (Ambion, Inc., Austin, TX). cDNA libraries were constructed and indexed with the TruSeq RNA Sample Preparation kit, and the average insert size was 361 bp (Illumina, Inc., San Diego, CA). Sixteen samples (four biological replicates at four time points) were sequenced on two randomly assigned lanes with the HiSeq2000 (Illumina, Inc., San Diego, CA). On average, 51.2 million paired‐end 100 nt reads were generated for each sample. We previously reported transcript abundance for 34 soybean aquaporin genes from a separate analysis of these reads (Locke & Ort, 2015).

### 2.3 Sequence alignment and processing

Raw reads were trimmed to remove adapter sequence contamination with Trimmomatic (Bolger, Lohse, & Usadel, 2014) and aligned to the soybean genome (Williams 82 assembly version 2 annotation version 1; (Schmutz et al., 2010)) in TopHat version 2.1.1 (Kim et al., 2013). Mapped reads per gene were counted with HTSeq‐count (www‐huber.embl.de/users/anders/HTSeq/; (Anders, Pyl, & Huber, 2015)).

### 2.4 RNA‐seq statistical analyses

Genes with very low or no expression (<8 counts for all biological replicates at every time point) were filtered from the data set prior to statistical analysis. Several normalization strategies were tested, of which log_2_‐transformed trimmed mean of M‐values (TMM) (Robinson & Oshlack, 2010) most effectively normalized the data for parametric analysis. TMM correction factors were calculated using the edgeR package (McCarthy, Chen, & Smyth, 2012; Robinson, McCarthy, & Smyth, 2009). The log_2_(TMM)‐normalized expression was analyzed by repeated‐measures ANOVA using PROC MIXED (SAS 9.4, SAS Institute, Cary, NC), in which time point was the main effect and plot was the repeated measures subject. This program was chosen because the repeated measures structure of the time course data set is not readily modeled by specialized statistical packages for gene expression. The residuals for each gene were tested for normality, a requirement for the validity of parametric testing; those genes with non‐normal residual error distributions were instead analyzed in a repeated‐measures ANOVA using simulated, bootstrapped “*F*”‐distributions. In total, 357 genes fell into this nonparametric testing category; these are noted in Supporting information Dataset S1. *p*‐values were corrected for false discovery rate (fdr) among all comparisons, and genes with fdr‐corrected *p*‐values below 0.05 and log_2_(fold change) ≥1 between at least two time points, which corresponds to a doubling or halving of transcription, were considered to be differentially expressed (DE). Log_2_(fold changes) for DE genes were calculated from log_2_(TMM) normalized counts for each time point (11:00/ZT5:20, 14:00/ZT8:20, 17:00/ZT11:20) relative to 8:00/ZT2:20.

Gene ontology (GO) annotations were downloaded from SoyBase (https://soybase.org/; (Grant, Nelson, Cannon, & Shoemaker, 2009)), and to identify GO terms that were overrepresented among DE genes, Fisher's exact test with a Bonferroni *p*‐value correction was applied through the tool on SoyBase (Morales et al., 2013). Dominant diurnal transcription patterns among DE genes were identified with *k*‐means clustering of log_2_(TMM) values in SAS PROC FASTCLUS.

To examine the differences in variance that could indicate differential environmental effects on diurnal transcription patterns, the coefficient of variation (CV) was calculated for each gene/time point (absolute value[*SD*/mean]). The CVs for the four time points were then averaged, and the genes were ranked based on average CV with the highest average CV indicating the most variability in transcript abundance. Tukey's fences were used to identify genes that were moderate outliers (mean CV > Q3 + [1.5 × (Q3‐Q1)]) and extreme outliers (mean CV > Q3 + [3 × (Q3‐Q1)]) for average CV. Additionally, the equality of CVs among time points was tested for each gene with an asymptotic test (Feltz & Miller, 1996) using the R package “cvequality”(Marwick & Krishnamoorthy, 2018). In this analysis, each time point (8:00/ZT2:20, 11:00/ZT5:20, 14:00/ZT8:20, and 17:00/ZT11:20) was treated as a group, and log_2_(TMM), the same variable used to test the equality of means in the repeated‐measures model, was the variable for which CV was calculated.

### 2.5 Chlorophyll analysis

Chlorophyll was extracted from tissue samples, and total chlorophyll content and chlorophyll *a*/*b* ratios were determined according to Porra, Thompson, and Kriedemann (1989) and Lichtenthaler (1987). Data were analyzed by repeated‐measures ANOVA in PROC MIXED (SAS 9.4, SAS Institute, Cary, NC).

### 2.6 GRN inference

A dynamic gene regulatory network (GRN) was inferred using a graphical Gaussian model with the GeneNet package v. 1.2.13 in R (Opgen‐Rhein & Strimmer, 2007; Schaefer, Opgen‐Rhein, & Strimmer, 2015). A very strict threshold was used to filter the DE gene list for GRN inference (fdr‐corrected *p *<* *0.0001, log_2_(fold change) ≥ 2), and 1,199 strongly DE genes were included in the analysis. Significant edges and directions with fdr‐corrected *p *<* *0.0001 were included in the GRN.

### 2.7 Accession numbers

Sequence data and read counts from this study can be found at the NCBI GEO data repository under accession number GSE114878.

## 3 RESULTS AND DISCUSSION

### 3.1 RNA‐seq output and clustering

mRNA sequencing yielded over 820 million reads from 16 samples. Sufficient reads were counted for 36,059 genes to include in the analysis (Supporting information Dataset S1), which represents 64% of the 56,044 protein‐coding genes predicted in the soybean genome (Schmutz et al., 2010). Of these, 11,984 genes were found to be differentially expressed [DE; fdr‐corrected *p *<* *0.05, log_2_(fold change ≥ 1)] between time points over the course of the day. The percentage of genes that were DE, approximately 21%, is smaller than the proportion of circadian‐regulated genes found in the Arabidopsis transcriptome (Blasing et al., 2005; Covington, Maloof, Straume, Kay, & Harmer, 2008), which probably reflects the shorter time course examined here. Additionally, for the 357 genes that had to be analyzed using nonparametric tests, there was a higher Type II error rate than among the 35,702 genes analyzed with parametric tests, due to the lower statistical power of the nonparametric tests. The analysis method for each gene, parametric or non‐parametric, is noted in Supporting information Dataset S1.

Varying environmental conditions over the four sampling days have the potential to introduce substantial variability in transcription. A microarray study comparing field and chamber‐grown rice (*Oryza sativa*) found that over 7,000 genes required a term to be included in a model that summarized environmental variables in order for the model to accurately predict transcription (Nagano et al., 2012). For the four RNA sampling days in the present study, temperature, PPFD, and RH were remarkably consistent (Supporting information Figure S1). RH showed the greatest variability, with early‐morning values ranging from <75% to >85%. Nonetheless, that this extensive DE gene list was able to be statistically resolved despite environmental variation among days implies a very robust diurnal response in these genes, especially considering the high potential for biological variation among the four replicates, which were spatially segregated field plots sampled on different days. Thus, the DE gene list presented and discussed in this study likely represents genes with the most consistent, environmentally stable diurnal responses.

**Figure 1:**
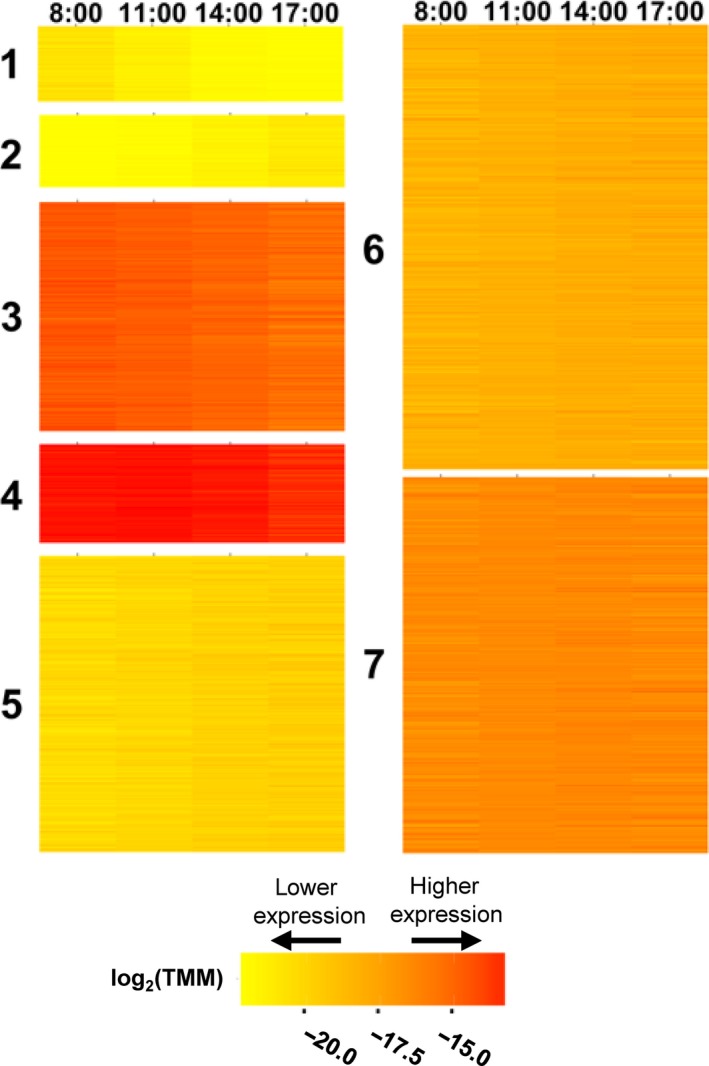
*k*‐means clustering of diurnally differentially expressed genes in soybean. Normalized expression values [log_2_(TMM)] are shown (yellow = low expression, red = high expression) for four time points [8:00 (ZT2:20), 11:00 (ZT5:20), 14:00 (ZT8:20), 17:00 (ZT11:20)] measured during daylight hours

Diurnal transcription patterns were examined by clustering DE genes based on log_2_(TMM) values (Figure 1). Each cluster contained between 692 and 3141 genes. Cluster analysis revealed seven distinct diurnal expression patterns (Figures 1, Supporting information Figure S2). Cluster 1 showed relatively low levels of expression in the morning that declined further throughout the day, whereas transcript abundance in cluster 2 was very low in the morning and increased through the day. Clusters 3 and 4 showed declining expression patterns throughout the day, although the overall magnitude of expression was greater in cluster 4. Clusters 5, 6, and 7 all increased slightly from 8:00/ZT2:20 to 11:00/ZT5:20 and 11:00/ZT5:20 to 14:00/ZT8:20 and seemed to plateau at 14:00/ZT8:20. However, they differed in overall magnitude, with 5 < 6<7.

Gene lists for each cluster were tested for overrepresentation of GO terms for each distinct diurnal expression pattern (Supporting information Dataset S2). No specific GO terms showed significantly higher representation in clusters 1 and 2. mRNA 5′‐UTR binding was the most overrepresented GO term in cluster 3, whereas Rubisco activator activity (i.e., Rubisco activase), was the most overrepresented GO term in cluster 4. Only one GO term, mitochondrial mRNA modification, was overrepresented in cluster 5. Protein import into the nucleus was the most highly overrepresented GO term in cluster 6, and cluster 7 showed the highest overrepresentation of the response to hormone stimulus GO term.

### 3.2 CV analyses

Coefficient of variation was significantly different among time points for 3,231 genes, which was 8.96% of those included in the analysis (Supporting information Dataset S1), indicating stronger environmental impacts at some time points for those genes. For these genes, large variation in expression at one or two time points may have prevented a gene from being identified as DE, despite a trend in the means. This effect may have been exacerbated for genes with one or more very low‐expression time points, due to the logarithmic transformation that was applied to TMM‐normalized data, which was necessary to shape the data in a normal distribution as is appropriate for ANOVA and post‐hoc tests.

The mean CV for each gene was calculated from the CVs of the four time points. Based on these mean CVs, 262 genes (0.7%) were extreme outliers for high mean CV (Supporting information Dataset S1), meaning that these genes had extremely high variation among sampling days relative to other genes. There were 964 genes (2.6%) considered as moderate outliers for high mean CV. As the lower outlier fence was below zero, no genes had a mean CV that fell below the lower outlier fence.

### 3.3 Known circadian genes

**Figure 2:**
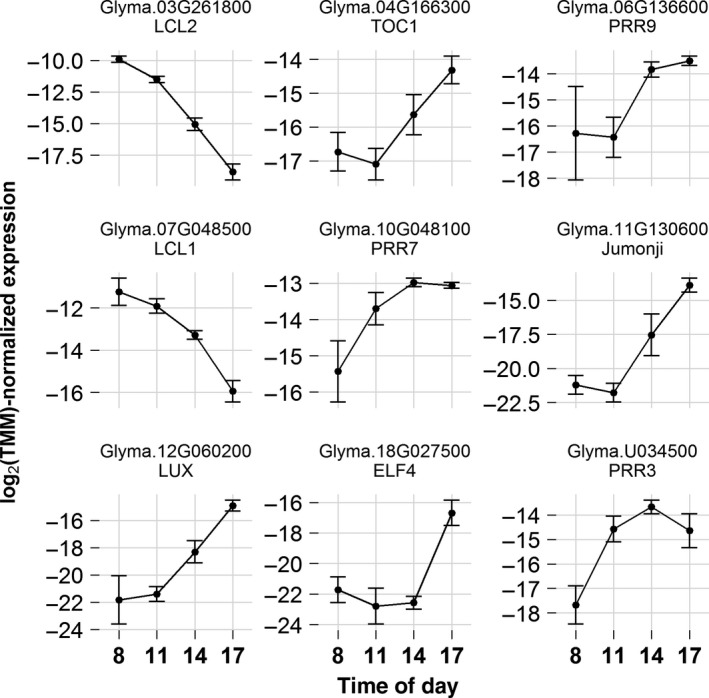
Diurnally differentially expressed circadian rhythm‐related genes in field‐grown soybean. Transcript abundance is shown as mean[log_2_(TMM)] ± *SD* for four time points [8:00 (ZT2:20), 11:00 (ZT5:20), 14:00 (ZT8:20), 17:00 (ZT11:20)] measured during daylight hours. *ELF4*,*EARLY FLOWERING 4*;*LCL1/2*,*LHY/CCA1‐LIKE 1*/*2*;*LUX*,*LUX ARRHYTHMO*;*PRR3/7/9*,*PSEUDO‐RESPONSE REGULATOR 3*/*7*/*9*;*TOC1*,*TIMING OF CAB EXPRESSION 1*

We first examined the expression of key circadian‐related genes in field‐grown soybean leaves during daylight hours (Figure 2). TIMING OF CAB EXPRESSION1 (TOC1), LATE ELONGATION HYPOCOTYL (LHY), and CIRCADIAN CLOCK ASSOCIATED1 (CCA1) are considered as universal components of the circadian clock in plants. CCA1 and LHY are transcription factors with high expression at dawn that repress genes that are more highly expressed in the evening, such as *TOC1*. *GmTOC1*,* GmLHY/CCA1‐LIKE 1* (*GmLCL1*), and *GmLCL2* are soybean circadian clock component genes with homology to *TOC1*,* LHY*, and *CCA1* genes in Arabidopsis, respectively. Thus, *GmLCL1* and *GmLCL2* also likely encode transcription factors that negatively regulate *GmTOC1* (Alabadí et al., 2001; Liu et al., 2009). *GmTOC1*,* GmLCL1*, and *GmLCL2* were DE in field‐grown soybean leaves, and their transcription patterns were consistent with their homologs in other species (Figure 2). *GmTOC1* was assigned to cluster 2, with low transcript abundance in the morning that increased sixfold by 17:00/ZT11:20. Transcription of *GmLCL1* and *GmLCL2* declined over the course of the day, and both of these genes were assigned to cluster 4 (Figure 2). The transcription patterns of these genes match the patterns measured for chamber‐grown soybean (Liu et al., 2009; Marcolino‐Gomes et al., 2014).

Expression of several other circadian‐regulated genes has been studied in chamber‐grown soybean (Marcolino‐Gomes et al., 2014). Therefore, we aimed to compare these previous findings to expression patterns from field‐grown soybean. The expression profiles of soybean genes *EARLY FLOWERING 4* (*GmELF4*), *GmJumonji*,* LUX ARRHYTHMO* (*GmLUX*), and *PSEUDO‐RESPONSE REGULATOR 3* (*GmPRR3*) from the controlled‐environment study were similar to those found in this study (Figure 2). However, *GmPRR7* and *GmPRR9* expressions stabilized or continued to increase after 14:00/ZT8:20 in the field (Figure 2), which differed from a sharp decline after 8 h of light in the chamber experiment, despite similar day lengths in the field (13:23–13:47 h) and in the chamber studies (14 h) (Marcolino‐Gomes et al., 2014). *GmGIGANTEA* (*GmGI*), showed a similar difference between field and chamber experiments (Supporting information Figure S3). Despite a trend in diurnal transcription, *GmGI* was not DE in this study, which is likely the result of high variability at 8:00/ZT2:20 and its non‐normal distribution of residual error, which required analysis with the lower power, nonparametric test. CV was also significantly different among time points for *GmGI* (Supporting information Dataset S1). In rice, *GI* was sensitive to temperature only during part of the diurnal cycle (Nagano et al., 2012); this differential sensitivity to environmental conditions could also influence the variability observed among time points for *GmGI*. Stresses such as drought may alter circadian‐regulated gene expression (Marcolino‐Gomes et al., 2014), and soybeans often encounter drought in the field. However, the expression of these circadian‐regulated genes (*GmPRR7*,* GmPRR9*, and *GmGI*) in the field did not match the patterns seen under growth chamber drought conditions. Thus, it is possible that diurnal gene regulation also depends on factors unique to field conditions that are not present in highly controlled environments. These results suggest that transcriptomic analyses conducted on soybeans grown in controlled environments may not fully represent the expression patterns of field‐grown soybean.

### 3.4 Photosynthesis: light absorption

**Figure 3:**
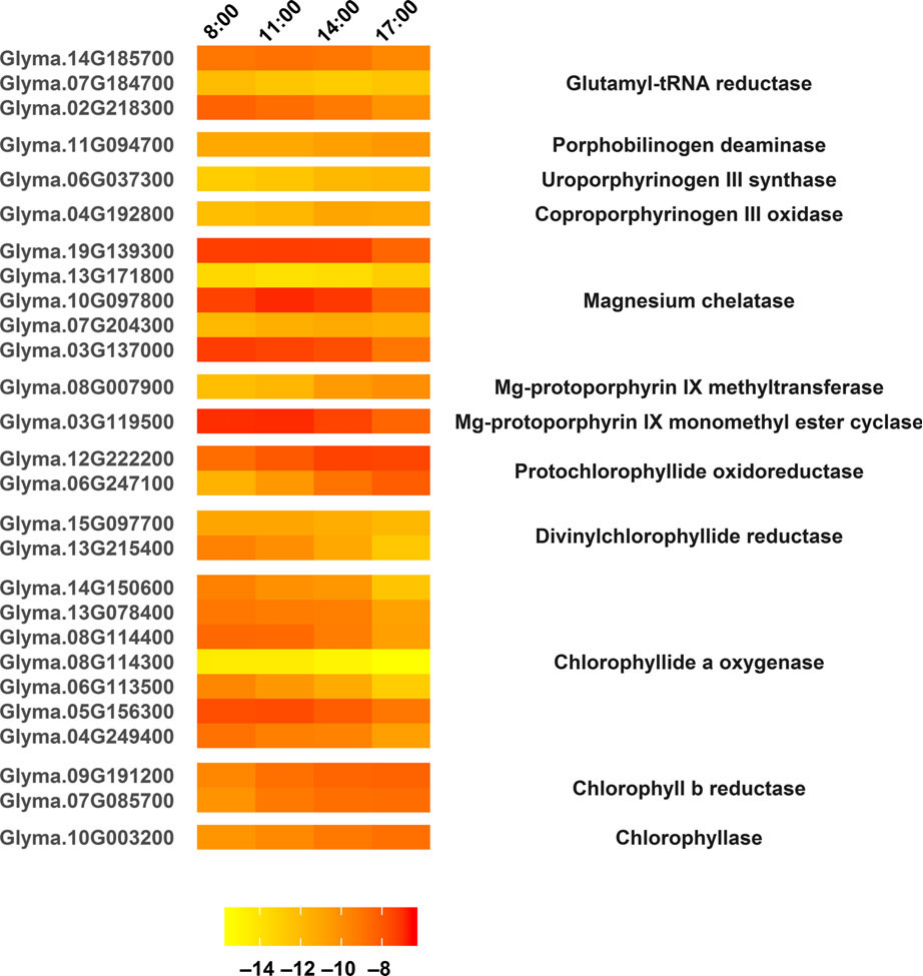
Diurnally differentially expressed genes within the chlorophyll biosynthetic pathway in field‐grown soybean. Gene loci are listed on the left, and the corresponding enzymes are listed on the right. Expression values [log_2_(TMM)] are shown (yellow = low expression, red = high expression) for four time points [8:00 (ZT2:20), 11:00 (ZT5:20), 14:00 (ZT8:20), 17:00 (ZT11:20)] measured during daylight hours

**Figure 4:**
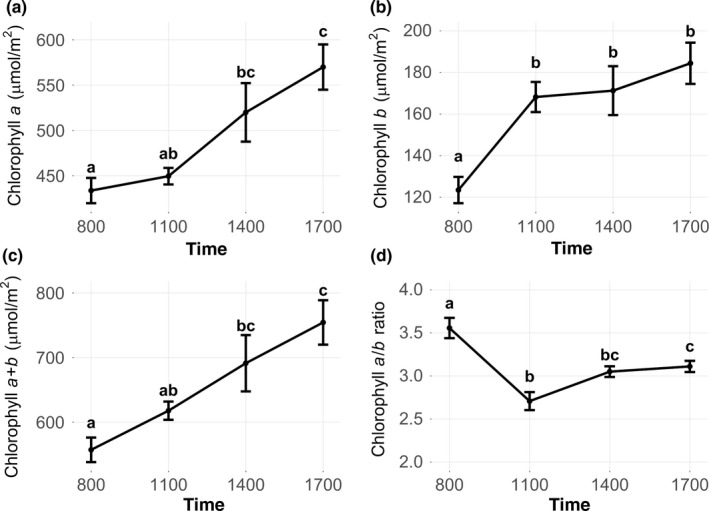
Diurnal chlorophyll contents and *a*/*b* ratios of field‐grown soybean. (a) Chlorophyll *a*, (b) chlorophyll *b*, (c) total chlorophyll content, and (d) chlorophyll *a*/*b* ratios were measured at four time points [8:00 (ZT2:20), 11:00 (ZT5:20), 14:00 (ZT8:20), 17:00 (ZT11:20)] during daylight hours in leaves from field‐grown soybean on DOY 228 in 2013. Values represent the means ± *SD* errors (*n* = 10). Different letters within panels represent significant differences (*p *<* *0.05)

Light absorption by chlorophyll is the first step in photosynthesis. GO terms for chlorophyll binding, chlorophyll catabolic processes, and chlorophyll biosynthetic processes were all significantly overrepresented in cluster 4, which included the most highly transcribed genes with diurnally decreasing transcription, so we further examined gene expression changes in the pathway for chlorophyll synthesis. Diurnal regulation of chlorophyll biosynthesis is light‐dependent and likely mediated by phytochrome signaling pathways (Ilag, Kumar, & Söll, 1994; Reinbothe & Reinbothe, 1996). The first steps of the pathway are common to synthesis of all tetrapyrroles (chlorophyll, heme, and siroheme). Most of the DE genes in this portion of the pathway, including genes encoding porphobilinogen deaminase, uroporphyrinogen III synthase, and coproporphyrinogen III oxidase, were upregulated as the day progressed (Figure 3), which coincided with increases in total chlorophyll and chlorophylls *a* and *b* throughout the day (Figure 4a–c). However, the highly transcribed genes for glutamyl‐tRNA reductase (GluTR), the rate‐limiting enzyme for tetrapyrrole biosynthesis, showed a large decline in expression at 17:00/ZT11:20 compared to 8:00/ZT2:20 (Figure 3). Circadian studies of chlorophyll gene expression in Arabidopsis have shown a rapid increase in GluTR transcripts occurring within 3 h of illumination and declining during the second half of the day under constant light intensity (Matsumoto, Obayashi, Sasaki‐Sekimoto, Ohta, & Takamiya, 2004), supporting other reports of light‐induced expression of genes encoding GluTR (Tanaka et al., 1996). While we did not see the same initial increase in expression in this study, it is possible that GluTR transcript levels had already peaked at or around the first measurement of the day (8:00/ZT2:20), which was approximately 2 h after sunrise, despite light intensity peaking around 13:00.

The insertion of Mg^2+^ into the protoporphyrin ring by magnesium chelatase (MgCh) is the first step in the chlorophyll‐specific branch of chlorophyll synthesis. This enzyme contains three subunits which are encoded by several genes, the expression patterns of which varied during daylight hours (Figure 3). Gene expression varies among MgCh subunits in Arabidopsis, which may be due to involvement of different subunits in different roles in binding protoporphyrin and signaling of the chlorophyll synthesis pathway (Matsumoto et al., 2004) and roles of specific subunits in other processes, such as abscisic acid (ABA) signaling (Du et al., 2012). For field‐grown soybean, expression of genes encoding successive enzymes in the chlorophyll branch [Mg‐protoporphyrin IX methyltransferase (MgMT) and protochlorophyllide oxidoreductase (POR)] increased as the day progressed, and transcript abundance was highest at 17:00/ZT11:20 (Figure 3). Although the four time points in this study may not capture the true peaks of transcription for most genes, it is likely that peak transcript abundance for MgMT and POR occurred between 14:00/ZT8:20 and 17:00/ZT11:20, which was consistent with POR expression in chamber‐grown Arabidopsis (Matsumoto et al., 2004). However, in chamber‐grown Arabidopsis, MgMT transcription peaks at midday. This pattern of transcription is unlikely to have occurred in the field‐grown plants, with solar midday at approximately 13:00 CDT, considering that the highest transcript abundance was measured at 17:00/ZT11:20, followed by 14:00/ZT8:20. Measured transcript abundance of the genes for Mg‐protoporphyrin IX monomethyl ester cyclase and divinyl chlorophyllide reductase was highest around 11:00/ZT5:20 and declined later in the day (Figure 3) as was seen in Arabidopsis (Matsumoto et al., 2004).

The final steps of chlorophyll synthesis constitute the chlorophyll cycle (Tanaka & Tanaka, 2007), in which chlorophylls *a* and *b* are interconverted by three enzymes, chlorophyllide *a* oxygenase (CAO), chlorophyll *b* reductase (CBR), and 7‐hydroxymethyl chlorophyll *a* reductase. This cycle serves to adjust the chlorophyll *a* to chlorophyll *b* ratio, which is thought to regulate light harvesting complex size and degradation (Tanaka, Kobayashi, & Masuda, 2011). CAO is putatively encoded by four genes in soybean and CBR by two genes in soybean [Phytozome (http://www.phytozome.net/soybean.php)], and the transcription of all of these decreased steadily from 8:00/ZT2:20 to 14:00/ZT8:20. As with GluTR, rapid increases in Arabidopsis CAO gene expression occur during the first 3 h of illumination (Matsumoto et al., 2004), so it is possible that CAO gene expression was highest at the 8:00/ZT2:20 measurement in this study. Transcription of the gene for chlorophyllase (Glyma.10G003200), which removes the phytol chain from chlorophyll *a* to form chlorophyllide *a* in the first step of chlorophyll catabolism, increased during daylight hours (Figure 3, Supporting information Dataset S1), but this enzyme may also be posttranslationally regulated (Harpaz‐Saad et al., 2007).

Since several genes in the chlorophyll biosynthesis pathway showed diurnal expression patterns (Figure 3) and chlorophyll content fluctuates on diurnal and circadian cycles in some plant species (Bukatsch & Rudolph, 1963), we measured diurnal chlorophyll content and *a*/*b* ratios in field‐grown soybean, albeit not from the same plants used for RNA‐seq analyses (Figure 4). Diurnal PPFD for the day on which chlorophyll was measured was similar to the days on which RNA samples were collected (Supporting information Figures S1, S4), with the exception of some possible cloud cover at 14:00/ZT8:20 on the chlorophyll sampling day. This lowered PPFD to approximately 750 μmol m^−2^ s^−1^ at 14:00/ZT8:20 on the chlorophyll sampling date, as compared to an average of approximately 1500 μmol m^−2^ s^−1^ at 14:00/ZT8:20 for the RNA sampling dates. Total chlorophyll content increased throughout the course of the day (Figure 4c). While both chlorophylls *a* and *b* increased from 8:00/ZT2:20 to 17:00/ZT11:20, the increase in total chlorophyll from 8:00/ZT2:20 to 11:00/ZT5:20 was largely due to an increase in chlorophyll *b* (Figure 4b), whereas total chlorophyll increased between later time points mostly due to an increase in chlorophyll *a* (Figure 4a). Thus, the chlorophyll *a*/*b* ratio changed significantly throughout the day, with a reduction occurring between 8:00/ZT2:20 and 11:00/ZT5:20 followed by a slight increase during the afternoon (Figure 4d). This pattern in soybean was similar to diurnal chlorophyll *a*/*b* ratios in wheat (*Triticum aestivum*; Busheva et al., 1991). While an earlier study reports no significant diurnal changes in total leaf chlorophyll content of soybean plants grown in greenhouses (Wickliff & Aronoff, 1962), a more recent study conducted in environmental growth chambers shows chlorophylls *a* and *b* in soybean leaves declining early during the light period and increasing after 4–8 h of illumination (Pan et al., 2015). Thus, by experiencing natural light conditions in the field, which are typically more intense and more varied than in growth chambers, soybean leaves in this study exhibited largely different patterns in diurnal chlorophyll content. These differing environmental conditions may also explain why gene expression was altered in our study compared to previous research in some chlorophyll synthesis‐related genes, as the natural fluctuations in light intensity and/or quality in the field may regulate expression differently compared to the timed, typically flat light regimes used in controlled environments.

Diurnal changes in chlorophyll content and *a*/*b* ratios did not always align with the expression patterns of genes involved in chlorophyll biosynthesis. While this may partially be due to environmental conditions on the different sampling dates (Supporting information Figures S1, S4), it is also likely due to the posttranslational regulation of key enzymes. While total chlorophyll increased throughout the day (Figure 4c), GluTR transcripts decreased (Figure 3). However, previous research shows no correlation between GluTR protein abundance and chlorophyll synthesis (Nogaj, Srivastava, van Lis, & Beale, 2005). Instead, GluTR activity is negatively regulated by accumulation of pathway products, such as hemes, Mg‐based rings, and divinyl protochlorophyllide *a* (Tanaka & Tanaka, 2007). In the present study, decreased CAO gene expression (Figure 3) was associated with a plateau in chlorophyll *b* content during the later three time points of daylight hours (Figure 4b), but evidence suggests that CAO's regulatory mechanisms are primarily posttranscriptional (Tanaka & Tanaka, 2011), so decreasing the transcription of CAO genes may not directly cause the shifts in chlorophyll *a*/*b* ratios of newly synthesized pigment. However, while posttranslational regulation may be a key for fine‐tuning rates of chlorophyll‐associated protein synthesis, the clear diurnal and circadian patterns associated with these genes indicate a role for diurnal regulation of transcription, even if the function is only to ensure sufficient protein levels for later fine‐tuning via posttranslational control.

### 3.5 Photosynthesis: energy transfer and electron transport

**Figure 5:**
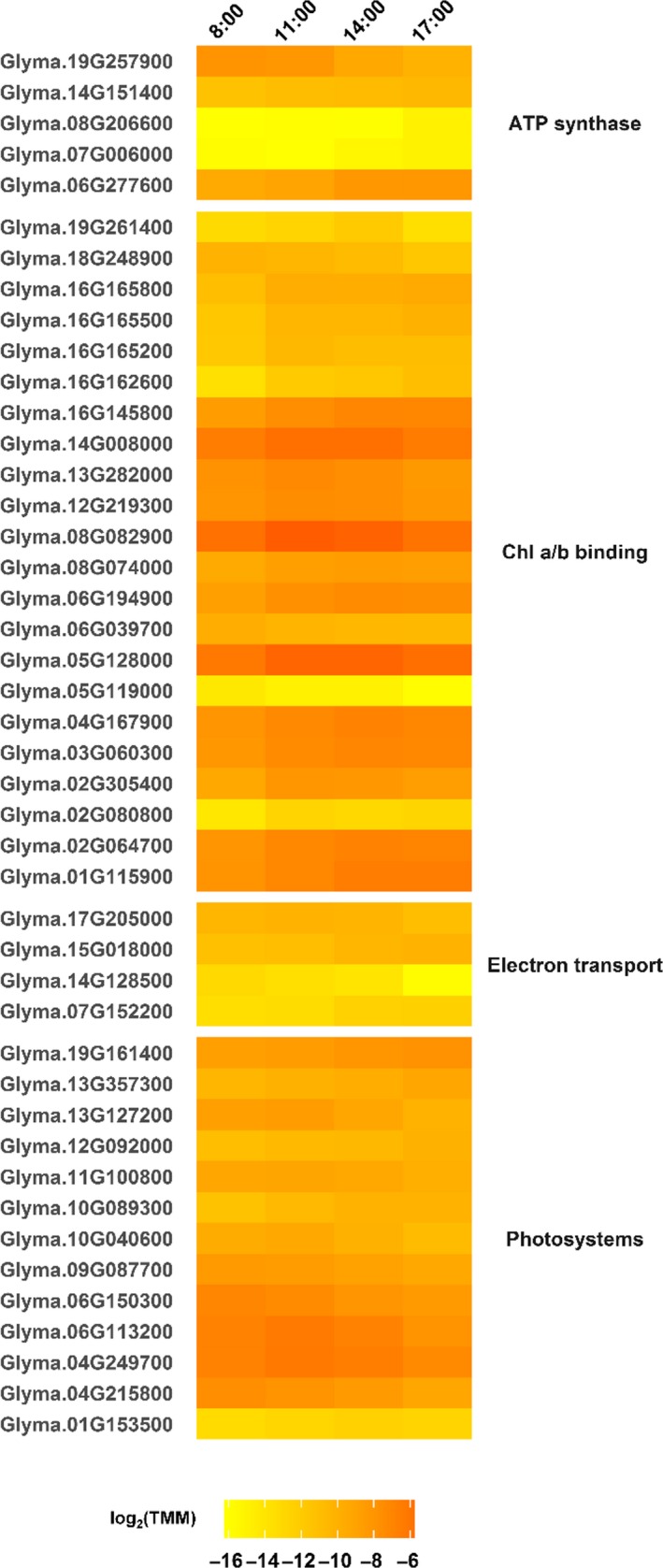
Diurnally differentially expressed genes related to photosynthetic energy transfer and electron transport in field‐grown soybean. Gene loci are listed on the left, and the corresponding protein functions are listed on the right. Expression values [log_2_(TMM)] are shown (yellow = low expression, red = high expression) for four time points [8:00 (ZT2:20), 11:00 (ZT5:20), 14:00 (ZT8:20), 17:00 (ZT11:20)] measured during daylight hours

Chlorophyll *a*/*b* binding proteins have diurnal transcription fluctuations in several species that tend to peak around midday (Martino‐Catt & Ort, 1992; Meyer, Thienel, & Piechulla, 1989). In field‐grown soybean, most genes encoding chlorophyll binding proteins showed lowest transcript abundance at 8:00/ZT2:20, with expression increasing throughout the day or peaking in the afternoon (Figure 5). This increase could be related to the concurrently increasing gene expression of many components of the chlorophyll synthesis pathway, including MgMT and POR (Figure 3), and increased chlorophyll content in leaves throughout the day (Figure 4c).

Transcription of photosystem (PS)‐related genes showed three distinct patterns. The most common pattern was low expression early and late in the day, with transcript abundance highest at either 11:00/ZT5:20 or 14:00/ZT8:20 (Figure 5). All of the genes with this pattern encoded proteins associated with PSII, including PsbD (or D2; Glyma.01G153500) and Psb28 (or PsbW; Glyma.10G040600, Glyma.13G127200). In wheat and Arabidopsis, *PsbD* shows similar circadian transcription patterns, with peak expression occurring during midday (Nakahira, Baba, Yoneda, Shiina, & Toyoshima, 1998; Noordally et al., 2013). While Psb28 is involved in PSII synthesis and assembly (Bečková et al., 2017; Dobakova, Sobotka, Tichy, & Komenda, 2008), it may also affect chlorophyll synthesis, which we found to change diurnally (Figure 4), and D1 synthesis (Dobakova et al., 2008; Mabbitt, Wilbanks, & Eaton‐Rye, 2014). The next most common transcription pattern for PS‐related genes was an increase in expression throughout the day (Figure 5), which included genes related to both PSI and PSII. The least common pattern was a decline in expression throughout the day (Figure 5), which was seen in the gene encoding PsaK (Glyma.09G087700), which may facilitate the interaction between light harvesting complex I and PSI (Jensen, Gilpin, Jurgen, & Scheller, 2000).

Expression of DEs related to other proteins and enzymes involved with electron transport and ATP synthase varied over the course of the day (Figure 5). Three of the four genes related to electron transport were involved with iron ion binding (Glyma.14G128500, Glyma.15G018000, Glyma.17G205000), but expression patterns differed for each gene.

### 3.6 Photosynthesis: C_3_ cycle

**Figure 6:**
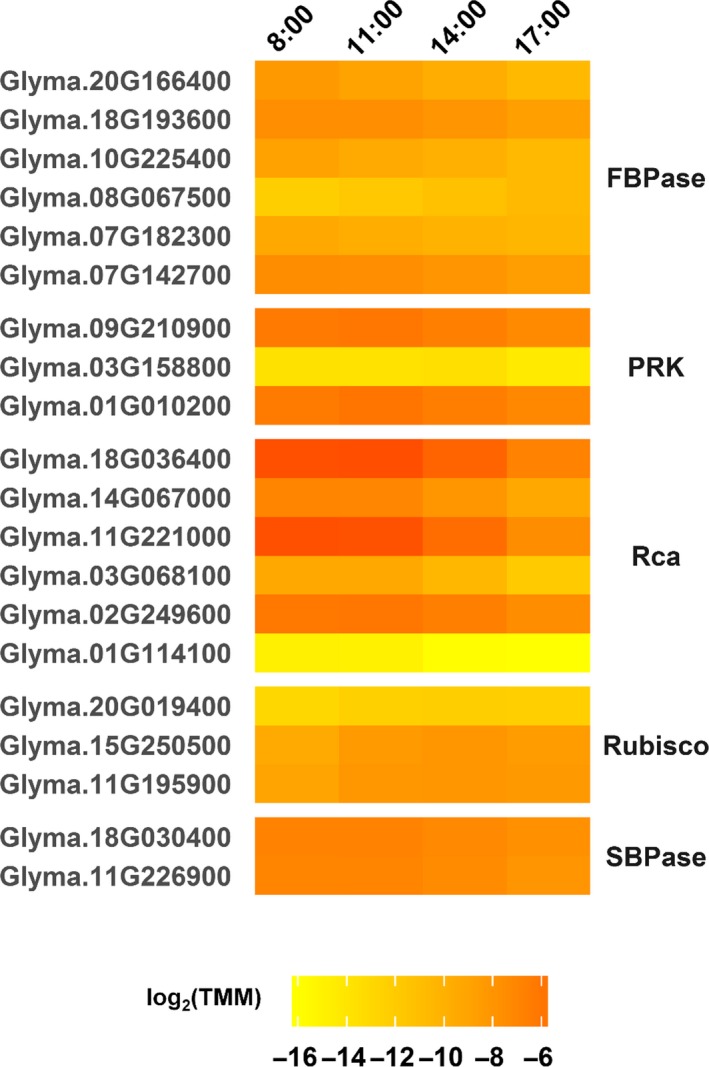
Diurnally differentially expressed genes related to key enzymes of the C_3_ cycle in field‐grown soybean. Gene loci are listed on the left, and the corresponding enzymes are listed on the right. Expression values [log_2_(TMM)] are shown (yellow = low expression, red = high expression) for four time points [8:00 (ZT2:20), 11:00 (ZT5:20), 14:00 (ZT8:20), 17:00 (ZT11:20)] measured during daylight hours. FBPase, fructose 1,6‐bisphosphatase; PRK, phosphoribulokinase; Rca, Rubisco activase; Rubisco, ribulose 1,5‐bisphosphate carboxylase/oxygenase; SBPase, sedoheptulose 1,7‐bisphosphatase

Key steps in the C_3_ cycle are catalyzed by the enzymes ribulose 1,5‐bisphosphate carboxylase/oxygenase (Rubisco), fructose 1,6‐bisphosphatase (FBPase), sedoheptulose 1,7‐bisphosphatase (SBPase), and phosphoribulokinase (PRK). Rubisco proteins are expected to have a half‐life of days, so diurnal transcriptional changes likely would not have much impact on protein abundance. Accordingly, none of the Rubisco small subunit‐encoding genes were DE in our analysis. Because poly‐A selection was performed on total RNA prior to RNA‐seq library construction, transcription of the chloroplast‐located Rubisco large subunit genes was not measured in our study. However, transcription of genes that encode Rubisco large subunit binding proteins (Glyma.11G195900, Glyma.15G250500, Glyma.20G019400) increased during the morning were highest at 14:00/ZT8:20 and then declined at 17:00/ZT11:20 (Figure 6). These binding proteins are involved in Rubisco holoenzyme assembly (Gutteridge & Gatenby, 1995), so their diurnal transcription could indicate a role for Rubisco complex assembly in diurnal adjustment of CO_2_ fixation capacity, as incident light levels often drop below photosynthetic saturation by 17:00/ZT11:20. Rubisco activase (Rca) is required for Rubisco activity, and expression of diurnally affected Rca genes was generally highest at 8:00/ZT2:20 and 11:00/ZT5:20 but decreased strongly at later time points (Figure 6), which is similar to the circadian‐regulated expression of Rca genes seen in Arabidopsis, apple (*Malus domestic*), rice, and tomato (*Lycopersicon esculentum*); (Liu, Taub, & McClung, 1996; Martino‐Catt & Ort, 1992; Pilgrim & McClung, 1993; To, Suen, & Chen, 1999; Watillon, Kettmann, Boxus, & Burny, 1993). Similar to Rca gene expression, diurnal transcription of FBPase‐, SBPase‐, and PRK‐encoding genes largely peaked in the morning and declined as the day progressed (Figure 6). The ferredoxin‐thioredoxin system contributes to posttranslational regulation of these enzymes, and circadian rhythms have been observed in the expression of thioredoxin‐type genes and proteins (Barajas‐López et al., 2011). Thus, multiple levels of diurnal and/or circadian regulation are involved in the C_3_ cycle. The decreased transcription of Rca genes and other C_3_ cycle‐related genes observed in this study, apparent as early as 14:00/ZT8:20, when diurnal photosynthesis typically peaks for field‐grown soybean (Rogers et al., 2004), might anticipate nightfall.

### 3.7 Photosynthesis: Carbon metabolism

**Figure 7:**
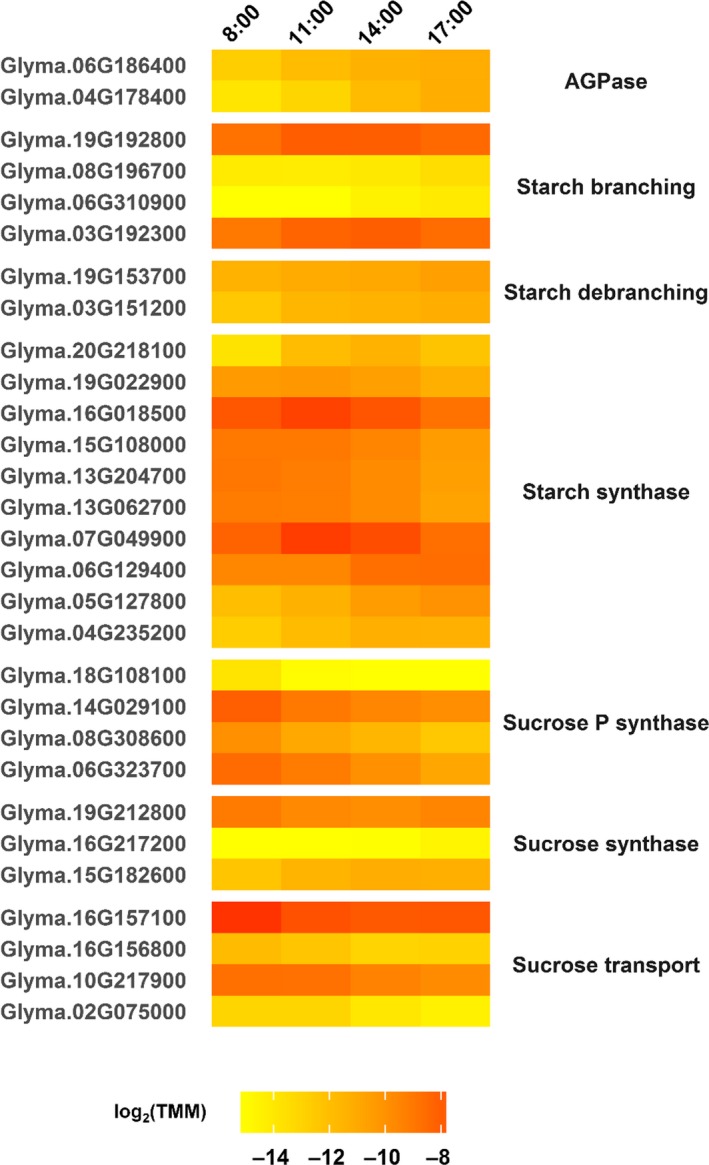
Diurnally differentially expressed genes related to starch and sucrose in field‐grown soybean. Gene loci are listed on the left, and the corresponding enzymes or classes of enzymes are listed on the right. Expression values [log_2_(TMM)] are shown (yellow = low expression, red = high expression) for four time points [8:00 (ZT2:20), 11:00 (ZT5:20), 14:00 (ZT8:20), 17:00 (ZT11:20)] measured during daylight hours. AGPase, ADP glucose pyrophosphorylase

Leaf carbohydrate pools fluctuate diurnally. Starch is synthesized during the day when light can drive photosynthesis and is broken down during the night to fuel cellular respiration, and this has been measured previously in field‐grown soybean (Rogers et al., 2004; Stitt & Zeeman, 2012). Starch degradation is strongly regulated by the circadian clock to ensure that starch stores last throughout the night (Graf & Smith, 2011), and several genes related to starch synthesis and breakdown were differentially regulated during the day in field‐grown soybean in this study. Genes encoding ADP glucose pyrophosphorylase (AGPase), which catalyzes the entry step to starch synthesis, increased in expression as the day progressed (Figure 7). AGPase gene expression increases in the presence of sucrose (Sokolov, Dejardin, & Kleczkowski, 1998), the level of which increases in field‐grown soybean during daylight hours (Rogers et al., 2004). However, other posttranscriptional modes of AGPase regulation lead to more fine‐tuned responses of AGPase during fluctuating conditions (Geigenberger, 2011), and some of these processes, including the ferredoxin‐thioredoxin system, have shown circadian rhythms (Barajas‐López et al., 2011). A variety of starch synthase transcription patterns has been observed in Arabidopsis (Smith et al., 2004). Starch synthase genes in field‐grown soybean also showed varied patterns of expression throughout the day, but expression was often highest at 8:00/ZT2:20 or 11:00/ZT5:20 and declined later in the day (Figure 7). Starch branching enzyme genes showed varied expression throughout the day, whereas expression of starch debranching‐related genes increased as the day progressed (Figure 7).

Sucrose is exported from source leaves to be used for energy and biomass production in sink leaves, roots, and developing seeds. Although sucrose synthase gene expression varied during the day, expression of genes related to sucrose phosphate synthase and sucrose transport largely declined during daylight hours. This is the opposite pattern of sucrose accumulation resulting from photosynthesis over the course of the day. However, if these enzymes have a half‐life of several hours, this could be indicative of the leaf anticipating the onset of darkness.

### 3.8 Gene regulatory network (GRN)

**Figure 8:**
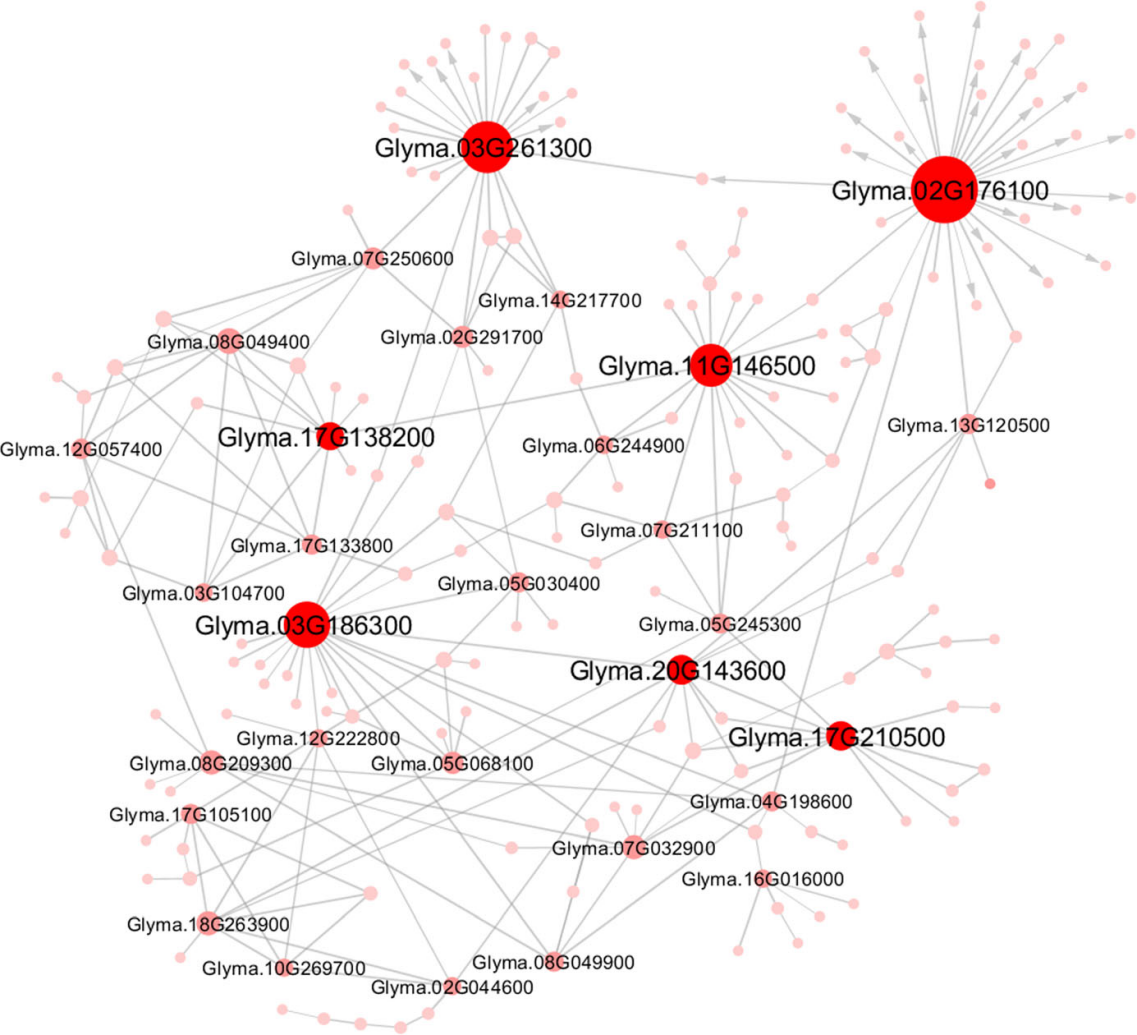
Gene regulatory network (GRN) analysis. Of the 1199 highly differentially expressed genes included in the analysis, 199 were assigned to the network (circles), which shows 267 significant “edges” (lines). Of these, 28 edges showed significant direction of causality (lines with arrowheads). Genes with ≥10 edges were considered “nodes” (large red circles). A very strict significance cutoff (fdr *p *<* *0.0001) was used for determining edges and directionality

To identify genes that are integral to diurnal cycles in soybean, a GRN was inferred using a very strict significance cutoff (fdr *p *<* *0.0001) for both edges and directionality. Among the 1,199 highly DE genes included in the analysis, 199 were assigned to the network, and 267 significant relationships or “edges” were inferred among those (Figure 8, Supporting information Dataset S3). *A priori* identification of transcription factors and/or binding sites was not included in the analysis, so the inferred edges do not necessarily indicate direct regulation of transcription between nodes. The direction of causality was significantly determined for 28 of those relationships. Seven genes or “nodes” had 10 or more connections (Figure 8, Table 1), which are likely important regulators of diurnal functions in field‐grown soybean. One of these nodes, *PSEUDO‐RESPONSE REGULATOR 5* (*GmPRR5*; Table 1), is known to be directly involved with circadian rhythms (Bendix et al., 2015; Fujimori, Sato, Yamashino, & Mizuno, 2005; Nakamichi, Kita, Ito, Yamashino, & Mizuno, 2005) and interacts with CONSTANS to promote flowering in response to day length in Arabidopsis (Hayama et al., 2017). Two transcription factors, YABBY (GmYABBY16; (Zhao et al., 2017)) and the ethylene‐responsive SHINE 2‐RELATED (SHN2), were also identified as hub nodes (Table 1). YABBY plays an important role in plant development, including determining organ polarity, such as adaxial versus abaxial surfaces of leaves, and may be involved in flowering and seed development. In soybean, expression of *GmYABBY16* and other *GmYABBY* genes is affected by drought and salt stress and ABA application (Zhao et al., 2017), and YABBY likely regulates other transcription factors important to soybean development (Shamimuzzaman & Vodkin, 2013). SHN transcription factors are involved in regulating cutin, suberin, and wax protein levels in Arabidopsis (Licausi, Ohme‐Takagi, & Perata, 2013; Shi et al., 2011), and the poplar (*Populus* spp.) SHN2 has been shown to regulate secondary cell wall formation in tobacco (*Nicotiana tabacum*) transformants (Liu et al., 2017).

**Table 1:** Hub nodes from gene regulatory network (GRN) inference analyses

Hub node genes	Protein annotation description^a^	Abbr.
Glyma.02G176100	Similarity (60%) with phosphoribosyl 1,2‐cyclic phosphate phosphodiesterase and ribonuclease z.	PHNP
Glyma.03G186300	Phosphatidylinositol‐4‐phosphate‐5‐kinase	PIP5K
Glyma.03G261300	Response regulator of two‐component system (PSEUDO‐RESPONSE REGULATOR 5)	PRR5
Glyma.11G146500	Aquaporin transporter	PIP2;4
Glyma.17G138200	YABBY protein	YABBY
Glyma.17G210500	Ethylene‐responsive transcription factor	SHN2
Glyma.20G143600	Leucine‐rich repeat N‐terminal domain/carbohydrate binding protein of the endoplasmic reticulum	LRRNT_2

a Obtained from SoyBase and/or Phytozome12 (JGI).

Several nodes were identified as genes encoding proteins involved in cellular processes. Phosphatidylinositol‐4‐phosphate‐5‐kinase (PIP5K) was identified as a hub node (Table 1) and is involved in vesicle‐mediated transport, cell adhesion, cell polarization, and cell migration (Irvine, 2003). In addition, water stress induces expression of specific PIP5Ks in Arabidopsis (Mikami, Katagiri, Iuchi, Yamaguchi‐Shinozaki, & Shinozaki, 1998). Leucine‐rich repeat N‐terminal 2 (LRRNT_2) was also identified (Table 1) and is a carbohydrate‐binding protein of the endoplasmic reticulum (Phytozome). The transcription of these genes was directly and negatively linked, although the direction of the interaction was not resolved. Similarly, the LRRNT_2 node was directly linked to SHN2 through a negative association, but again, the direction of repression was not evident. Another node, Glyma.02G176100, encoded a protein with 61% similarity to both a ribonuclease Z protein and phosphoribosyl 1,2‐cyclic phosphate phosphodiesterase (Table 1).

The final hub node was a gene encoding an aquaporin, PLASMA MEMBRANE INTRINSIC PROTEIN2;4 (GmPIP2;4), that acts as a channel and regulates water flux through the plasma membrane (Table 1). Our RNA‐seq analysis showed higher transcription of this gene at all time points compared to 8:00/ZT2:20, with the highest transcript abundance at 14:00/ZT8:20. Aquaporins show diurnal expression cycles in roots (Clarkson et al., 2000; Takase et al., 2011; Vandeleur et al., 2009) and may help regulate diurnal leaf water status in soybean leaves (Locke & Ort, 2015). As we reported previously, the diurnal transcription of *GmPIP2;4* in these leaves tracked atmospheric vapor pressure deficit, but its diurnal transcription pattern was inverse to the diurnal pattern of leaf hydraulic conductance (Locke & Ort, 2015). The GRN analysis indicated that *GmPIP2;4* expression influences or is influenced by many other genes, including a positive interaction with the YABBY node, although the direction of the relationship with *GmYABBY16* was unclear. As ABA treatment and salt stress upregulate but drought stress downregulates *GmYABBY16* expression (Zhao et al., 2017), this result is not surprising but deserves further analysis.

## 4 CONCLUSION

This diurnal picture of the field‐grown soybean transcriptome illustrates that genes in pathways either contributing to or depending on photosynthesis are often differentially transcribed over the course of the day. In addition, several genes act as hubs for regulating diurnal processes, either by directly regulating transcription of other genes or by integrating diurnal signals to control a key response (e.g., leaf water transport) with rapid and extensive downstream effects. Improving photosynthesis is becoming an increasingly important goal, as climate change and population growth test the limits of our agricultural production capacity. This data set can serve as a basis for further investigation of diurnally responsive pathways and eventual identification of genes and transcription factors that diurnally regulate photosynthesis and transpiration. If it is possible that diurnal variations in transcription of photosynthesis‐regulating genes could be manipulated, this could greatly contribute to improving photosynthesis in light‐limited conditions and water use efficiency at high midday vapor pressure deficit, and to better acclimate to changing climate. In addition, our comparisons with transcriptomic analyses conducted on soybean grown in controlled environments suggest that caution should be used when extrapolating gene expression patterns from controlled environments to field environments.

## AUTHORS’ CONTRIBUTIONS

AML and DRO designed the research; AML performed the research and analyzed the data; AML and RAS interpreted the data and wrote the paper with contributions from DRO.
