# Supplementary material for: Field‐grown soybean transcriptome shows diurnal patterns in photosynthesis‐related processes
Source: Plant Direct. 2018 Dec 4;2(12):e00099. doi: 10.1002/pld3.99 (PMC6508813; doi:10.1002/pld3.99)
Supplement: Supplementary file 1 [file PLD3-2-e00099-s001.pdf]

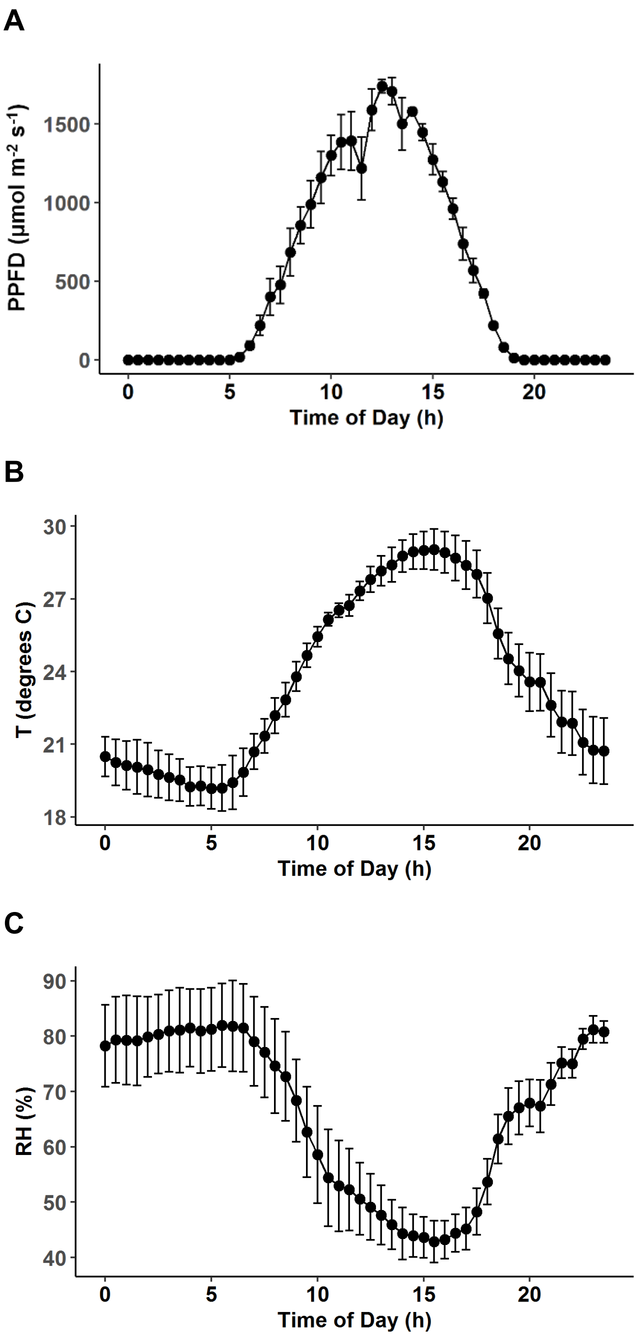

Supplemental Figure 1. Climate data from the 2010 RNA sampling dates. A) Photosynthetic photon flux density (PPFD), B) temperature (T), and C) relative humidity (RH) are shown as a function of time of day. Data represent means ± SE of the four sampling dates (August 14, 16, 18, 24) and were obtained from the University of Illinois Energy Farm.

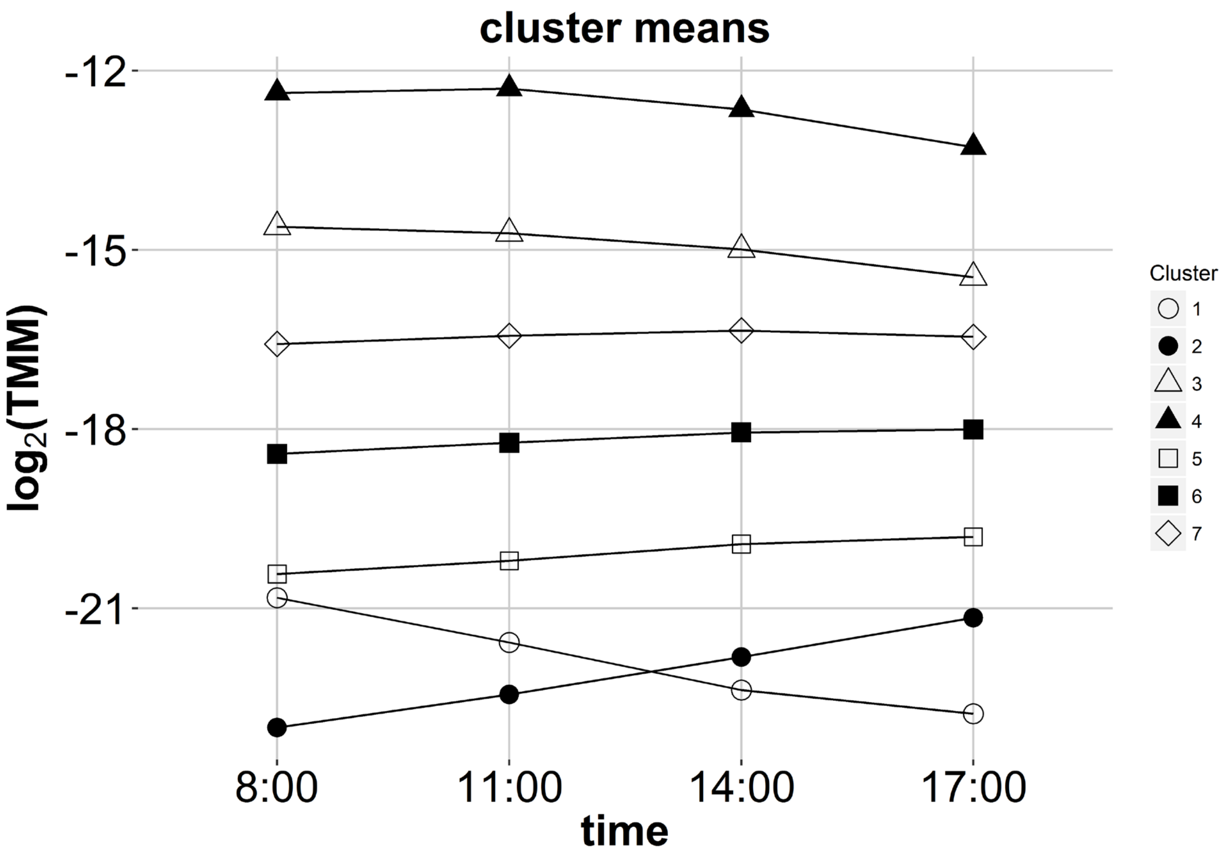

Supplemental Figure 2. Cluster means of diurnally differentially-expressed genes in soybean. Expression values [$\log_2$(TMM)] are shown for four time points (8:00/ZT2:20, 11:00/ZT5:20, 14:00/ZT8:20, 17:00/ZT11:20) measured during daylight hours. Values represent the means (n=4).

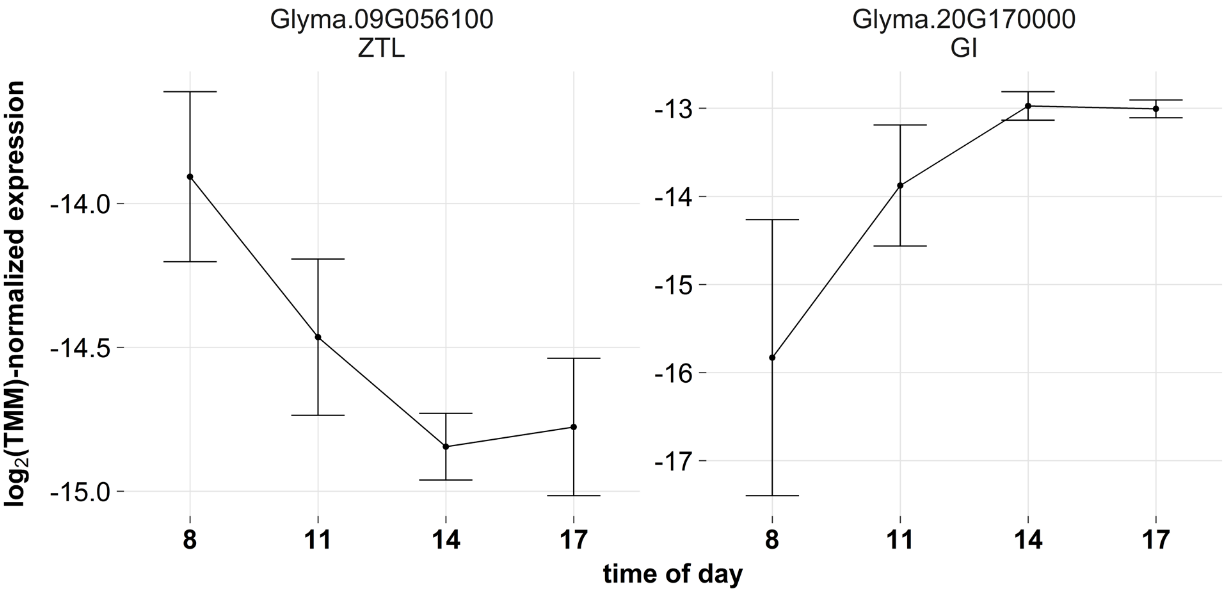

Supplemental Figure 3. Diurnally non-differentially-expressed circadian rhythm-related genes in field-grown soybean. Transcript abundance is mean[$\log_2$(TMM)] +/- standard deviation for four time points [8:00 (ZT2:20), 11:00 (ZT5:20), 14:00 (ZT8:20), 17:00 (ZT11:20)] measured during daylight hours. GI, GIGANTEA; ZTL, ZEITLUPE.

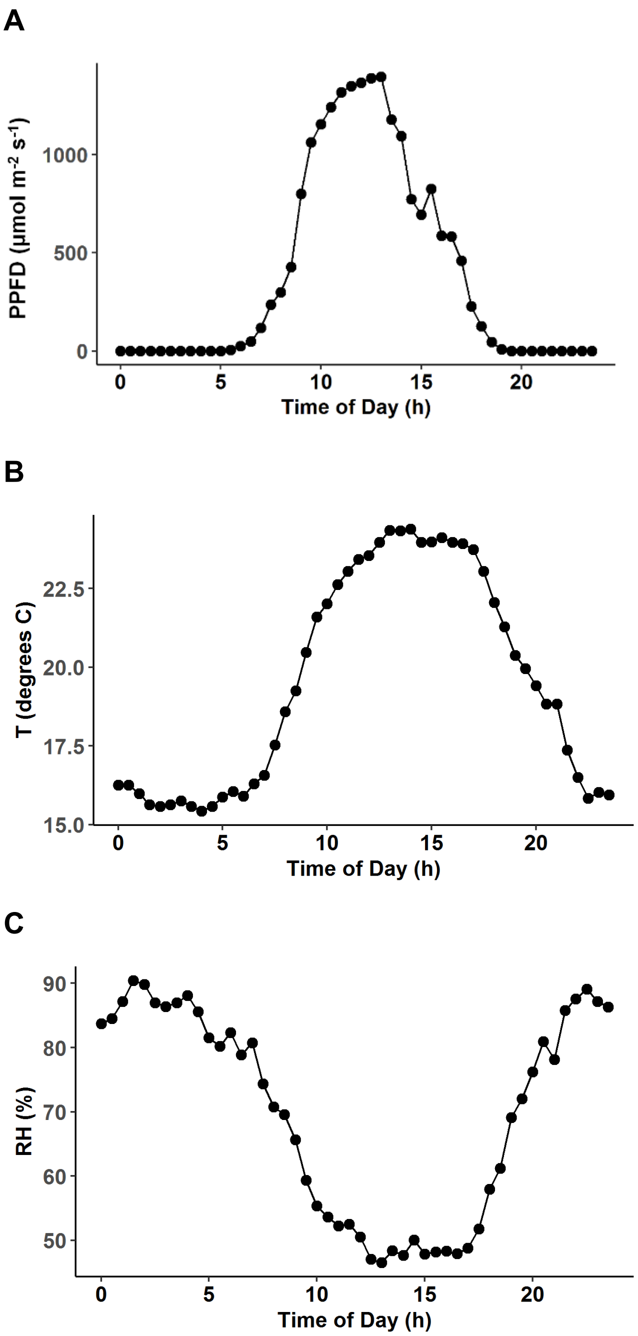

Supplemental Figure 4. Climate data from the chlorophyll sampling date in 2013. A) Photosynthetic photon flux density (PPFD), B) temperature (T), and C) relative humidity (RH) are shown as a function of time of day for 16 August. Data were obtained from the University of Illinois Energy Farm.
