# Supplementary material for: Field‐grown soybean transcriptome shows diurnal patterns in photosynthesis‐related processes
Source: Plant Direct. 2018 Dec 4;2(12):e00099. doi: 10.1002/pld3.99 (PMC6508813; doi:10.1002/pld3.99)
Supplement: Supplementary file 5 [file PLD3-2-e00099-s005.pdf]

-- Reviewer comments:
Reviewer #1:

The authors have thoughtfully addressed all of the concerns from the first review. The identification of a subset of genes that show significant variation presents some interesting avenues for follow up investigation into the nature of this variation. The dataset presented in this manuscript is an excellent resource for the community and will likely inspire additional studies.

I have two minor suggestions for improving consistency in the manuscript.

1. The dataset 1 has color shading in the spreadsheet that matches the original heatmap colors. It is very difficult to read with the dark blue and I'm wondering if the colors can be changed to the current yellow/red scheme that is now being used.

We appreciate that the reviewer caught this oversight in our revised manuscript. The heat map colors in Supplemental Data Set 1 have been changed to match the heat maps in the figures.

2. It is often instinctual to put the time of day in terms of hours rather than ZT but this requires the reader to remember what time of day dawn occurred. To avoid this unnecessary confusion, it is helpful to use ZT because it is always based on hours after dawn and a much more effective way of conveying time of day. Either have both included in the text or replacing the hours with ZT time will benefit the reader.

Thanks to the reviewer’s suggestion, we have improved the clarity of the manuscript by adding ZT to every mention of time throughout the manuscript text and figure legends. Both times are included at each mention, to maintain clarity for researchers who work primarily in circadian regulation as well as researchers who primarily work with crops in the field.
